# Development of a Multiplex PCR Assay for Efficient Detection of Two Potential Probiotic Strains Using Whole Genome-Based Primers

**DOI:** 10.3390/microorganisms11102553

**Published:** 2023-10-13

**Authors:** Despoina E. Kiousi, Dimitrios M. Karadedos, Anastasia Sykoudi, Panagiotis Repanas, Christina S. Kamarinou, Anthoula A. Argyri, Alex Galanis

**Affiliations:** 1 Department of Molecular Biology and Genetics, Faculty of Health Sciences, Democritus University of Thrace, 68100 Alexandroupolis, Greece; dkiousi@mbg.duth.gr (D.E.K.); dimikara61@mbg.duth.gr (D.M.K.); anastasia.g.sykoudi@gmail.com (A.S.); panrepa@hotmail.com (P.R.); christinakamarinou7@gmail.com (C.S.K.); 2 Institute of Technology of Agricultural Products, Hellenic Agricultural Organization—DIMITRA, 14123 Lycovrissi, Greece; anthi.argyri@gmail.com

**Keywords:** bacterial identification, strain-specific, lactic acid bacteria, probiotics, whole genome sequencing, comparative genomics, multiplex PCR

## Abstract

Probiotics are microorganisms that exert strain-specific health-promoting effects on the host. Τhey are employed in the production of functional dairy or non-dairy food products; still, their detection in these complex matrices is a challenging task. Several culture-dependent and culture-independent methods have been developed in this direction; however, they present low discrimination at the strain level. Here, we developed a multiplex PCR assay for the detection of two potential probiotic lactic acid bacteria (LAB) strains, *Lactiplantibacillus plantarum* L125 and *Lp. pentosus* L33, in monocultures and yogurt samples. Unique genomic regions were identified via comparative genomic analysis and were used to produce strain-specific primers. Then, primer sets were selected that produced distinct electrophoretic DNA banding patterns in multiplex PCR for each target strain. This method was further implemented for the detection of the two strains in yogurt samples, highlighting its biotechnological applicability. Moreover, it can be applied with appropriate modifications to detect any bacterial strain with available WGS.

## 1. Introduction

Probiotics are viable microorganisms that, when administrated in sufficient quantities, confer health benefits on the host [1]. They mainly display strain-specific immunomodulatory, antiproliferative, or antimicrobial activities [2] and are commonly employed for the management of gastrointestinal disorders [3] or as potential therapeutics against extraintestinal diseases [4]. Probiotics are available to consumers in fermented foods or supplements. Manufacturers are required to ensure the correct labeling of these products; specifically, the strains contained should be clearly stated, while their concentration should also be disclosed. Additionally, stable populations of probiotic bacteria should be guaranteed throughout production, storage, and distribution [5]. In this context, several molecular methods have been developed for the detection, identification, and monitoring οf probiotic microorganisms, including multilocus sequence typing (MLST) [6], pulsed-field gel electrophoresis (PFGE) [7], amplified ribosomal DNA restriction analysis (ARDRA) [8], and random amplified polymorphic DNA (RAPD) assay [9]. In addition, multiplex PCR assays with primers designed by comparative sequence analysis of polymorphic regions of conserved, housekeeping genes, such as 16S rDNA, *tuf* and *tufA* coding for the elongation factor EF-Tu, and *rpsL* coding for the 30S ribosomal subunit protein S12 have also been described [10,11,12]. However, the above approaches are labor-intensive, time-consuming, and display limited discrimination at the strain level [13,14].

The increased availability and accessibility to whole genome sequences of both cultured and uncultured strains has facilitated strain identification by genome-wide analysis of unique polymorphic regions [15,16]. Based on this, we sought to develop a comprehensive and robust multiplex PCR-based method for the identification of two potential probiotic LAB strains, *Lp. plantarum* L125 and *Lp. pentosus* L33, in monocultures and/or in complex samples, exploiting their recently published WGS [17,18]. Both strains that were previously isolated from fermented meat products [19] present desirable probiotic potential and biotechnological applicability, as they display adhesion capacity and antiproliferative effects in epithelial colon cancer cells [17,18], antimicrobial activity against human enteropathogens [20], as well as effectiveness as adjunct cultures for sausage fermentation [21]. The methodology presented can also be applied with appropriate modifications for the identification of any bacterial strain with available WGS in multiplex PCR.

## 2. Materials and Methods

### 2.1. Phylogenetic Analysis

To investigate the phylogenomic relationships and sequence identity between strains of different species, a phylogenetic tree based on the WGS of strains used in this study (*Lp. plantarum* L125, *Lp. pentosus* L33, *Lacticaseibacillus paracasei* SP5, *Lc. casei* ATCC 393, *Lc. rhamnosus* GG), reference genomes (*Levilactobacillus brevis* LMT1-73, *Latilactobacillus sakei* CBA3614, *Limosilactobacillus reuteri* 2010, *Ligilactobacillus salivarius* LPM01), and of the outgroup strain *Staphylococcus aureus* NCTC 8325, was constructed following a previously published method [22]. Briefly, the WGS of strains were downloaded from the NCBI genome database to be subsequently aligned using progressiveMauve [23]. The resulting phylogenomic tree file was visualized using the iTOL server [24]. The sequence identity of *Lp. plantarum* L125 and *Lp. pentosus* L33 to that of members of their species with deposited WGS was calculated using the Python module pyANI (Average Nucleotide Identity, ANI) [25]. Only assemblies at the scaffold/chromosome level were used for the analysis, and thus, 33 strains were collected for the *Lp. pentosus* species and 211 for the *Lp. plantarum* species (as of May 2023).

### 2.2. In Silico Pipeline for the Detection of Unique Regions in the Genome Sequence of Lp. plantarum L125 and Lp. pentosus L33

The WGS of *Lp. plantarum* L125 (accession number: JAIGOE000000000.1) and *Lp. pentosus* L33 (accession number: JAHKRU000000000.1) and raw sequencing reads were downloaded from the NCBI Assembly database. Raw sequencing reads were aligned against the genome of strains of the same species using Bowtie2 version 2.5.1 [26]. In more detail, an index containing the sequences was created using the command “bowtie2-build”, and then the command “bowtie2” was entered to produce an end-to-end sequence alignment in a SAM file format. Then, the command “samtools view” of the package SAMtools [27] was used to identify reads in the genome of *Lp. plantarum* L125 or *Lp. pentosus* L33 that did not align with sequences derived from other strains of the species (unique regions). Subsequently, the SAM file was converted to a BAM file; reads were sorted using “samtools sort”, and the BAM file was finally converted to two FASTQ files (one for the reverse and one for the forward strand reads) using the “samtools bam2fq” command. Finally, the unique reads were assembled into contigs using SPAdes version 3.13.1 [28]. Contigs were filtered based on their length, and only contigs with a length of >1000 bp were utilized for primer design. This length cut-off was applied to enable the design of PCR primers that can generate DNA products of variable length.

### 2.3. Design of Strain-Specific Primers

The sequence of the selected contigs was blasted against the “RefSeq Genome Database” and “Nucleotide collection” NCBI databases [29], using the following parameters: in the fields “Organism” and “Program selection” the categories “bacteria (taxid:2)” and the algorithm “Somewhat similar sequences (blastn)” were entered, respectively. Based on the results, contigs were selected on the basis of their identity with other deposited sequences; specifically, contigs containing regions with low alignment score (<40) to sequences derived from other bacteria and with high alignment score (≥200) for the strains of interest (*Lp. plantarum* L125 or *Lp. pentosus* L33) were selected for primer design. Finally, annotation of the contigs with Prokka [30] and PHASTER [31] was performed to verify the location of the unique genomic sequences. Primer design was performed using Primer-BLAST [32] with the following parameters; “PCR template”: the unique genome sequences generated by the in silico pipeline; Max Τm difference = 1; Database = Refseq representative genomes; Organism = bacteria (taxid:2); Primer specificity stringency = 6 total mismatches to unintended targets; at least 5 total mismatches within the last 5 bps at the 3′ end, ignore targets with 9 or more mismatches to the target; “Primer Size”: Min = 20; Opt = 22; Max = 25; “Primer GC content (%)”: Min = 45.0 and Max = 55.0. The specificity of the primers was, finally, examined against the “nr” and “Refseq representative genomes” NCBI databases, and their characteristics were investigated with the tool “PrimerDimer” of PrimerSuite [33]. Selected primer sets had a dG value of <−5. The dG value was also calculated between primers of different primer sets that would be included simultaneously in the same multiplex PCR reaction.

### 2.4. Bacterial Cultures and DNA Extraction

The strains used in this study are presented in Table 1. All lactobacilli were cultured in De Man, Rogosa, and Sharpe broth (MRS, Condalab, Spain) under static, anaerobic conditions at 37 °C for 16 h. Whole gDNA extraction was performed using the NucleoSpin Tissue kit (Macherey-Nagel, Düren, Germany), following the manufacturer’s instructions. The quantity and quality of the genomic DNA were determined spectrophotometrically (Thermo Scientific NanoDrop 1000 Spectrophotometer). DNA integrity was examined electrophoretically (1% *w*/*v* agarose, 70 V, 1 h).

### 2.5. Preparation of Yogurt Products Containing Lp. plantarum L125 or Lp. pentosus L33, Bacterial Sampling and DNA Extraction

Yogurts were prepared using pasteurized and homogenized bovine milk that was heated at 80 °C for 30 min and cooled to 45 °C. Then, an inoculum of a starter culture consisting of *Streptococcus thermophilus* and *L. bulgaricus* (CH-1, Chr. Hansen, Hørsholm, Denmark) and strains *Lp. plantarum* L125 or *Lp. pentosus* L33 were simultaneously added, as previously described [36]. Therefore, two different yogurts were produced: one containing *Lp. pentosus* L33 and starter culture and one containing *Lp. plantarum* L125 and starter culture. Briefly, LAB cells were harvested by centrifugation (6000× *g*, 5 min, 4 °C) and resuspended in milk to a final population of approximately 8 log CFU/mL. Milk samples were fermented in appropriate conditions (42 °C, 6 h) until the pH value reached 4.6, and then, yogurt samples were stored at 4 °C. Microbiological analysis ensued at two timepoints: immediately after the fermentation process and after 30-day storage at 4 °C (end of storage). For the microbiological analysis, one gram of each yogurt sample was serially diluted in Ringer’s solution (LABM, Lancashire, UK), spread on MRS agar (Condalab), and incubated anaerobically at 37 °C for 48 h (Anerocult C, Merck, Darmstadt, Germany). The plates corresponding to the concentration of 5 or 6 log CFU/g were used for DNA extraction. Briefly, all colonies were collected from agar plates using sterilized Ringer’s solution (LABM). Genomic DNA was extracted using the NucleoSpin Tissue kit (Macherey-Nagel), following the manufacturer’s instructions.

### 2.6. Multiplex PCR Assay Design and Gel Electrophoresis

Multiplex PCR assays were designed to enhance the discriminatory capacity of the assay at the strain level by combining 4 primer sets in each reaction to produce a distinct electrophoretic fingerprint for the strains of interest. PCR reactions were performed at a final volume of 20 μL and consisted of 5 units of Taq DNA polymerase (Minotech, Heraklion, Greece), 10 mM of each dNTP (Jena Bioscience, Jena, Germany), 1.5 mM MgCl_2_ (Minotech), 1× Taq polymerase buffer (Minotech), and 10 ng DNA template. Primers were added at a final volume of 4 μL and a final amount of 25 pmol in each reaction. The universal bacterial primer set P1/P2 was used in all multiplex reactions as a positive control [37]. Amplifications were carried out in the Veriti thermocycler (Applied Biosystems, Waltham, MA, USA), using the following conditions: 94 °C (1 min), followed by 25 cycles of 94 °C (45 s); 58 °C (30 s); and 72 °C (1 min), followed by a final extension step at 72 °C (10 min). The PCR products were separated on 2% (*w*/*v*) agarose gels, visualized under UV illumination, and photographed with a digital camera (Gel Doc EQ System, Bio-Rad, Hercules, CA, USA).

## 3. Results

### 3.1. Phylogenomic Analysis

**Figure 1:**
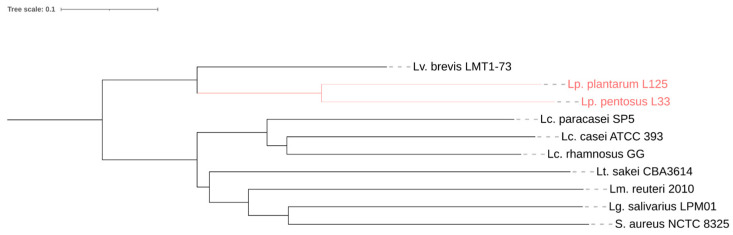
Phylogenomic tree containing *Lp. plantarum* L125 and *Lp. pentosus* L33 (highlighted in red) and other closely- or distantly related lactobacilli. The tree was constructed based on whole genome sequence alignment using progressiveMauve and was visualized on the iTol server.

A phylogenomic tree based on the WGS of closely and distantly related strains with the two bacteria of interest was constructed to determine their phylogenetic relationships (Figure 1). As expected, *Lp. plantarum* L125 and *Lp. pentosus* L33 that belong to the *Lactiplantibacillus* genus are closely associated, clustering together, while more distantly associated with the former *Lactobacillus casei* group (now known as the *Lacticaseibacillus* genus), as well as strains usually found in fermented meat products, including *L. sakei* or in association with the host, such as *L. reuteri* (Figure 1).

**Figure 2:**
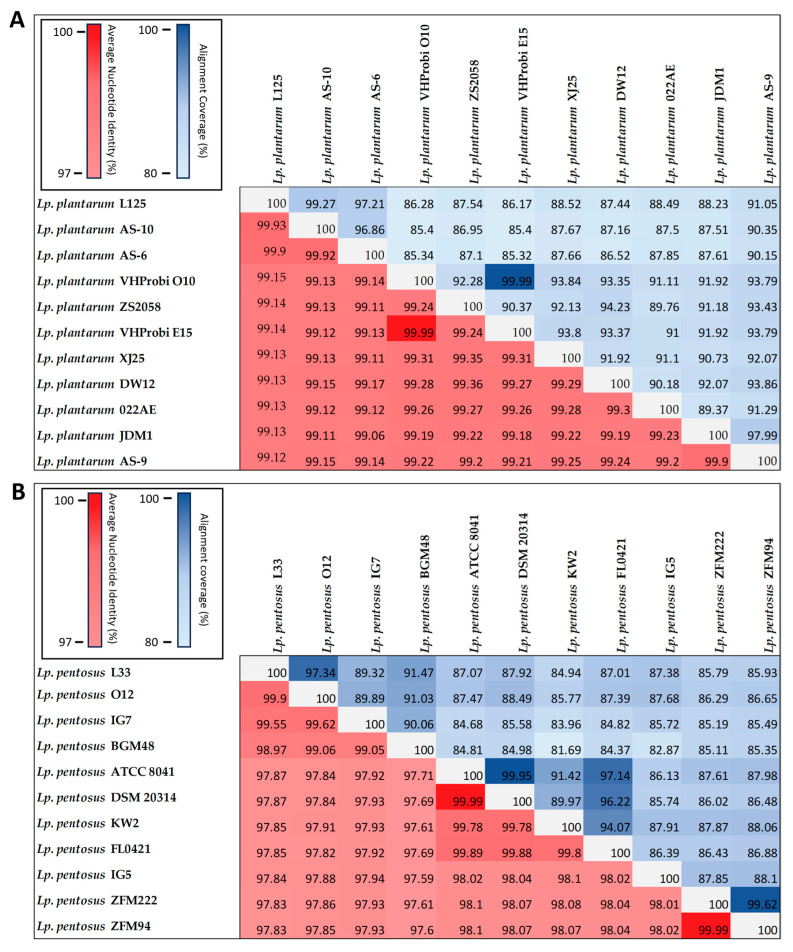
ANI (%) and alignment coverage (%) of *Lp. plantarum* L125 (**A**) and *Lp. pentosus* L33 (**B**) with members of the corresponding species.

Concerning the genome identity of the strains with other members of the species, ANI analysis was performed using the available WGS of strains at the chromosome/scaffold assembly levels (as of May 2023). It was shown that the strains shared high similarity with other members of the species (>98%) (Table S1). Of note, *Lp. plantarum* L125 presents an ANI score of 99.9% with strains *Lp. plantarum* AS-10 and *Lp. plantarum* AS-6, both isolated from fruit and vegetables, while *Lp. pentosus* L33 presents a sequence identity of 99.9%, with *Lp. pentosus* O12, a strain recently isolated from fermented table olives [38] (Figure 2).

### 3.2. Detection of Strain-Specific Unique Regions in the Genome of Lp. plantarum L125 and Lp. pentosus L33

The assembly of unique regions resulted in the construction of 31 contigs in the case of *Lp. plantarum* L125 and of 105 contigs for *Lp. pentosus* L33. Contigs were blasted individually against the NCBI databases “RefSeq Genome Database” (refseq_genomes) and “Nucleotide collection” (nr/nt), and contigs that presented high identity score (≥200) to the WGS of the strains and low identity score (<40) to other bacteria were selected for further analysis (Tables S2 and S3). More specifically, in the case of *Lp. plantarum* L125, four contigs satisfied these criteria and were selected for primer design. Six contigs showed high identity scores with the WGS of the strain and with other members of the species (*Lp. plantarum* AS-10, *Lp. plantarum* AS-6, *Lp. plantarum* BGAN8, *Lp. plantarum* M19) and of the closely related *Levilactobacillus brevis* G430 and the more distantly related *Liquorilactobacillus nagelii* AGA58, and 21 showed very high identity scores with strain *Lp. plantarum* L125 and multiple different bacteria (Table S2). These 27 contigs could represent highly conserved regions between different bacteria and were, therefore, excluded from further analysis. In the case of *Lp. pentosus* L33 no contig presented high identity with only the WGS of the strain, but rather all exhibited high similarity with the genome of the very closely related *Lp. pentosus* O12 strain (Table S3). Thus, contigs presenting high similarity to the genome of only one or two other bacteria (n = 5) were selected for primer design.

### 3.3. Design of Strain-Specific Primers for Lp. plantarum L125 and Lp. pentosus L33

Following the identification of putative unique sequences in the genome of the strains, strain-specific primers were designed using Primer-Blast. Four contigs were used as templates for *Lp. plantarum* L125 and five for *Lp. pentosus* L33. In total, 28 primer sets were designed for *Lp. plantarum* L125 and 42 for *Lp. pentosus* L33 (Table S4). Then, specific primer sets were selected to be tested in vitro in a multiplex PCR assay based on their capacity to produce a distinct electrophoretic pattern for the strains of interest (Table 2). Regarding the specificity of the primers, unintended products may be primarily found in bacteria not commonly found co-habiting with *Lp. plantarum* L125 or *Lp. pentosus* L33, while the lengths of these products are significantly different than those of the specific products (Table S5). Genome annotation showed that the regions amplified by the specific primers do not contain prophages and that they span non-coding and coding sequences (Table 3).

### 3.4. Validation of the Specificity of Primers In Vitro Using DNA Extracted from Monocultures or Fermented Dairy Products

**Figure 3:**
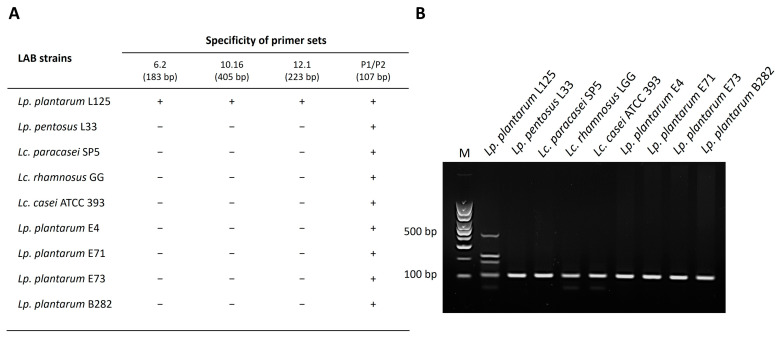
Tetraplex PCR assay for the detection of *Lp. plantarum* L125 with primer sets designed using the novel pipeline: (**A**) Specificity of primer pairs used in the tetraplex PCR. The expected product sizes are indicated; (**B**) Electrophoretic profile generated with the three specific primer sets and the universal bacterial primer set P1/P2 in tetraplex PCR with gDNA derived from *Lp. plantarum* L125 or other LAB. M: 100 bp DNA ladder.

**Figure 4:**
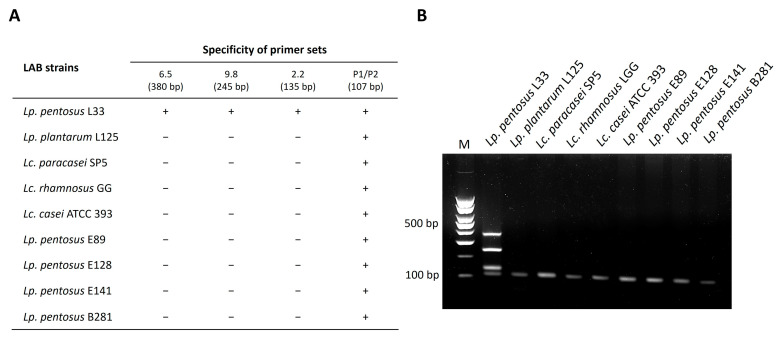
Tetraplex PCR assay for the detection of *Lp. pentosus* L33 with primer sets designed using the novel pipeline: (**A**) Specificity of primer pairs used in the tetraplex PCR. The expected product sizes are indicated; (**B**) Electrophoretic profile generated with the three specific primer sets and the universal bacterial primer set P1/P2 in tetraplex PCR with gDNA derived from *Lp. pentosus* L33 or other LAB. M: 100 bp DNA ladder.

**Figure 5:**
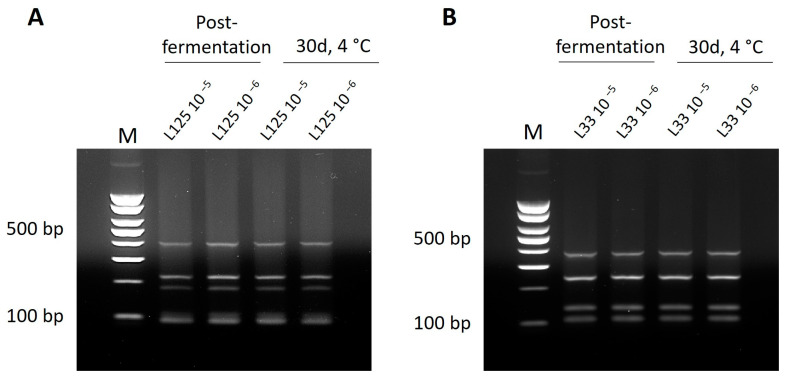
Identification of *Lp. plantarum* L125 (**A**) or *Lp. pentosus* L33 (**B**) in yogurt samples post- fermentation and after 30d storage at 4 °C, at two different concentrations (5 or 6 log CFU/g), via tetraplex PCR. M: 100 bp DNA ladder.

To determine the capacity of this pipeline to be used as an accurate and sensitive means to detect bacteria of interest, a multiplex PCR assay was designed using the selected primer sets. DNA from distantly and closely related strains was isolated and used as templates in the reactions. As shown in Figure 2 and Figure 3, the primer sets produce a distinct fingerprint only for the strains of interest. More specifically, an electrophoretic fingerprint consisting of 405, 223, and 183 bp bands is observed for *Lp. plantarum* L125 (Figure 3) and of 380, 245, and 135 bp bands for *Lp. pentosus* L33 (Figure 4), respectively. On the contrary, the other closely or distantly related strains only produce a PCR product derived from the universal primer set P1/P2 (positive control marker) (Figure 3 and Figure 4). Finally, to investigate the robusticity of this pipeline, multiplex PCR reactions were performed in DNA templates derived from yogurt samples inoculated with *Lp. plantarum* L125 or *Lp. pentosus* L33. As shown in Figure 5, the unique electrophoretic pattern is conserved for *Lp. plantarum* L125 and *Lp. pentosus* L33.

## 4. Discussion

**Figure 6:**
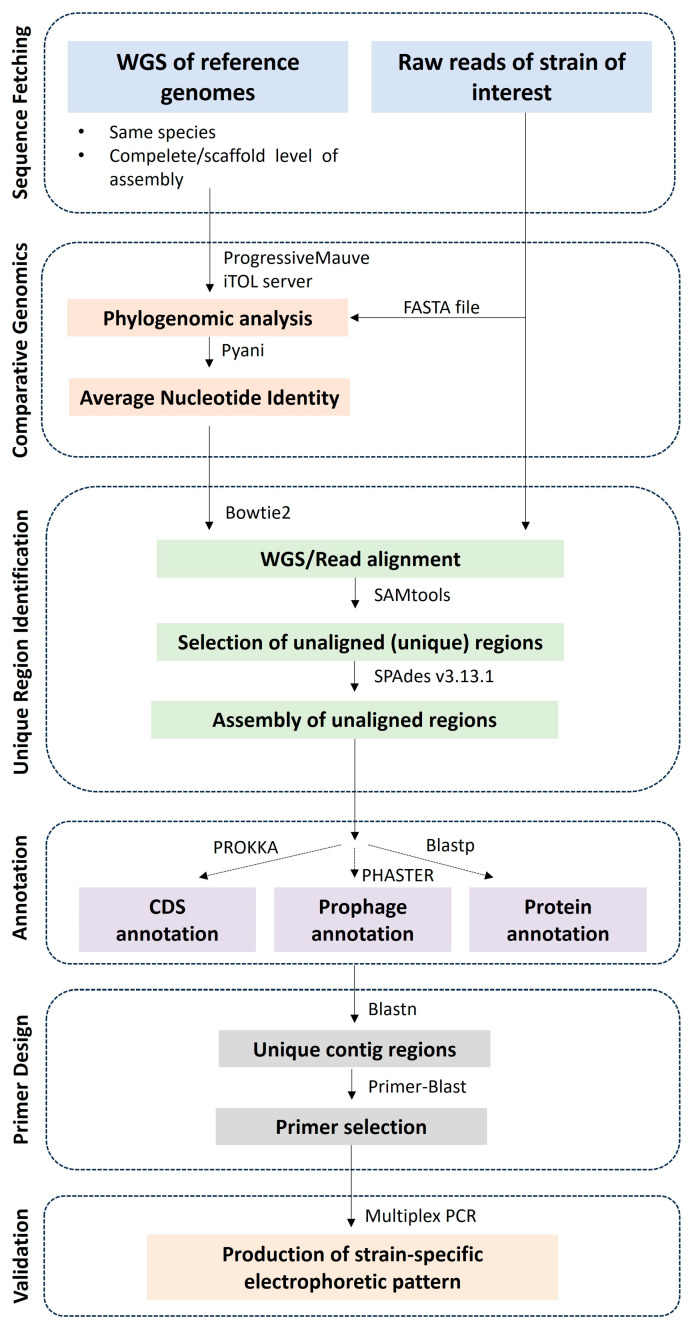
Schematic representation of the novel multiplex PCR-based methodology.

In this research article, we described a multiplex PCR assay to detect two potential probiotic strains, *Lp. plantarum* L125 and *Lp. pentosus* L33, in monocultures and food products. The methodology is presented in Figure 6 and can be followed to detect any other strain with available WGS. In more detail, raw sequencing reads of strains of interest are fetched from genome databases. Genomes of higher levels of assembly and, thus, quality are preferred; here, we included complete genomes and genomes at the scaffold level. Then, comparative genomics tools were utilized to investigate phylogenomic relationships and ANI between the strains of interest and other members of the species. In a recent, elegant study, comparative genomics has also been used for the development of a real-time PCR assay for efficient detection of *Lp. plantarum* group species in food samples [39]. This method is useful for species identification (inter-species discrimination) but cannot be applied to distinguishing individual strains (intra-species discrimination) [39]. In the case of our study, we found that both strains present high ANI (>99%), highlighting the need for automated pipelines to identify polymorphic genomic regions. Obviously, manually pinpointing the nucleotide differences between strains with such high genome identity would be an impossible task. Consequently, the sequence reads of the strains of interest are aligned against the genome of strains belonging to the same species to identify unique (unaligned) regions. These regions are subsequently assembled in an artificial chromosome and are blasted against all available bacterial sequences in the “nucleotide collection (nr/nt)” and “RefSeq Genome Database (refseq_genomes)” databases. This step is necessary to ensure that no unintended products will be detected in strains that belong to different species that could co-habit with the strains of interest in a complex matrix. It should be noted that this pipeline relies on the use of available datasets, and thus, it is inherently limited by the completeness of the databases it utilizes. The regions were filtered based on sequence identity, and unique sequences were annotated and used for primer design. The pipeline results in primers that can anneal to any sequence in the genome of the strains. Of note, prophage regions [16] or ORFs [40] were exclusively used for primer design in previous studies. Primer specificity is determined in silico using publicly available algorithms, including Primer-Blast. In the context of this study, unintended products were detected for some primer sets that are, however, of significantly different lengths or in bacteria derived from different ecological niches (Table S5). Finally, the primer sets that can generate a distinct electrophoretic pattern for the strains are selected for in vitro validation. Additionally, a universal gene should be added to the reactions as a positive control to validate the success of the amplification reactions. Here, we used the P1/P2 primer set that anneals to the V1 region of the universal bacterial gene 16S rDNA [37].

This method can be readily applied to the fermented food industry. Indeed, we managed to reproduce the same distinct electrophoretic pattern using gDNA derived from yogurt samples containing the strains of interest at different timepoints post-fermentation. The rapid, efficient, and accurate detection of bacterial strains during production, storage, and distribution is of great interest in the fermented food industry, as the distinct organoleptic characteristics of fermented dairy and non-dairy products are derived from the unique composition of the microbial matrix. Indeed, the communities that participate in fermentation determine texture, taste, and aroma and, ultimately, foodstuff quality and identity [41,42]. Furthermore, in the case of probiotic foods, specific strains are responsible for the favorable outcomes of their consumption, and thus, detection and monitoring of their population is required [5]. The availability of multi-omic platforms and the multi-level study of their effects on the host have provided evidence for their capacity to exert strain-specific activity [43,44,45]. Accordingly, an equally important aspect of bacterial identification in the context of fermented foods is monitoring for contaminant strains in the food matrix. Detection at the strain level can give definite evidence for the presence of spoilage strains [16] and discriminate against members of the same species with no harmful activity while also providing hints at appropriate decontamination methods. In this vein, we have previously shown that contaminant *Loigolactobacillus backii* strains are resistant to heat stress but may be sensitive to pressure treatment, resulting in more efficient decontamination approaches in the brewing industry [46]. Importantly, the novel method developed does not require a high level of technical expertise or sophisticated equipment; it can, therefore, be easily employed in the setting of the fermented food industry. Quantitative results can be generated from this pipeline using microbiological dilutions for culturable strains or fluorescent probes for quantitative PCR detection. Notably, functional foods are required to contain a viable count of at least six log CFU/g or mL [47]. Here, we employed a standard microbiological procedure to determine the capacity of the method to identify the bacteria of interest in yogurt samples, simulating established methodologies of the fermented food industry and ensuring the viability of the strains in the yogurt matrix after fermentation and in storage conditions. Appropriate tinkering can also facilitate quantification via RT-qPCR, as previously shown by Hernandez et al. (2020) [40]. Future work will aim at modifying the method to facilitate rapid, in situ detection with isothermal reactions, including loop-mediated isothermal amplification (LAMP) [48]. Nucleic acid isothermal reactions are today used on-site, mainly for pathogen detection during outbreaks or in the food chain, where no access to expensive, complex laboratory equipment is available. Therefore, harvesting this technology can simplify the pipeline and streamline strain detection in multiple settings.

This pipeline is versatile due to the fact that it can be employed for the identification of any strain with available WGS in complex matrices, therefore presenting a multitude of potential applications in basic and applied research. Community dynamics, the crosstalk between strains in undefined environmental samples, including the human host or experimental communities, is a field that has gained a lot of traction [49]. Metataxonomics have been used to track large-scale changes in the composition of microbial communities after exposure to different carbon sources [50], antibiotic or non-antibiotic drugs [51,52], host factors [53], or contamination of an existing microecosystem with extrinsic bacteria [54]; however, this approach is not appropriate for monitoring specific strains. Shotgun metagenomics could be used to pinpoint different strains of interest; however, our proposed method is quicker and quotes at a fraction of the cost, while results are straightforward and easier to interpret. Furthermore, strain-specific probes can find application in the visualization of host colonization patterns in situ. Indeed, elegant studies showed that bacteria may present strain and host-specific colonization patterns that could affect consumer physiology and microbiota homeostasis [44,45]. Furthermore, these probes can be useful in the study of the spatiotemporal interactions of complex microbial communities using (live) confocal and/or confocal high-content microscopy. Future work will focus on the use of this novel pipeline for the design of strain-specific probes for the quantitative and qualitative study of microbial interactions in complex matrices.

## 5. Conclusions

Strain-specific bacterial identification in the fermented food industry is a challenging yet necessary task. Available culture-dependent and culture-independent methods present limited discriminatory capacity at the strain level. Hence, we developed a multiplex PCR assay for efficient and rapid detection of two potential probiotic strains, *Lp. plantarum* L125 and *Lp. pentosus* L33, in monocultures and yogurt samples. Unique regions in the genome of the strains were detected via comparative genomic analysis and were used for primer design. A total of 28 primer pairs were designed for *Lp. plantarum* L125 and 42 for *Lp. pentosus* L33, among those, three primer sets were selected for each bacterium based on their capacity to produce a distinct electrophoretic pattern in a multiplex PCR. The method was successful in discriminating between the strains of interest and other closely or distantly related lactobacilli. Additionally, we managed to detect the strains in yogurt samples post-fermentation and after 30-day storage at 4 °C at two different concentrations (five and six log CFU/g), suggesting the biotechnological applicability of the method. The methodology developed can be followed with appropriate modifications to detect any bacterial strain with available WGS.

## Figures and Tables

**Table 1:** Lactobacilli used in this study.

Strain Name	Isolation Source	Available WGS	Reference
*Lp. plantarum* L125	Fermented sausages	Yes	[19]
*Lp. pentosus* L33	Fermented sausages	Yes	[19]
*Lc. paracasei* SP5	Kefir grains	Yes	[34]
*Lc. rhamnosus* GG	Commercial strain	Yes	DSMZ (Braunschweig, Germany)
*Lc. casei* ATCC 393	Commercial strain	Yes	ATCC (LGC Standards, Middlesex, UK)
*Lp. pentosus* B281	Fermented table olives	No	[35]
*Lp. pentosus* E89	Fermented table olives	No	[35]
*Lp. pentosus* E128	Fermented table olives	No	[35]
*Lp. pentosus* E141	Fermented table olives	No	[35]
*Lp. plantarum* B282	Fermented table olives	No	[35]
*Lp. plantarum* E4	Fermented table olives	No	[35]
*Lp. plantarum* E71	Fermented table olives	No	[35]
*Lp. plantarum* E73	Fermented table olives	No	[35]

**Table 2:** Primer sets utilized in multiplex PCR reactions to produce a strain-specific fingerprint for *Lp. plantarum* L125 and *Lp. pentosus* L33.

Primer Code	Primer Sequence (5′-3′)	Primer Length (bp)	Tm (°C)	GC Content (%)	Product Length (bp)
***Lp. plantarum* L125**					
6.2F	CCCGATAGAGGTTCTTCAAGCC	22	60.48	54.55	183
6.2R	ACTCCAAGGATCCAAACAAGCC	22	60.82	50.00	
10.16F	CGATTGCAGCAACGATAGATCC	22	59.84	50	405
10.16R	TAGACCCATTTTGCCAAGGTC	21	58.2	47.62	
12.1F	AGGAGCAATGTGATTCTACCAC	22	58.12	45.45	223
12.1R	AGGCAATGCTATCGTCCATGA	21	59.58	47.62	
***Lp. pentosus* L33**					
2.2F	CATATCGTCAACAATCCCACGG	22	59.46	50	135
2.2R	TAGCACTGTGGCTGAGTATTGG	22	60.09	50	
6.5F	TACTTTCTGATCTGGTCGGGTC	22	59.24	50.0	380
6.5R	GCTTTACCGGACATCCTCAATG	22	59.39	50.0	
9.8F	TGTTTTGGGTATAGCTGTGGC	21	58.56	47.62	245
9.8R	CGAACTCGGGCTAGAAATCATC	22	58.95	50	

**Table 3:** Annotation of genomic regions of *Lp. plantarum* L125 and *Lp. pentosus* L33 used for primer design.

Contig	Range of Primer Design	Prokka Annotation	Range of CDS	Blastp Annotation	Prophage Region
***Lp. plantarum* L125**					
Contig 6	515–697	Hypothetical protein	425–1276	Glycosyl-transferase	No
Contig 12	1908–2130	Hypothetical protein	1983–2468	Hypothetical protein	No
Contig 10	1947–2351	General stress protein A	1547–2557	Glycosyl-transferase	No
***Lp. pentosus* L33**					
Contig 6	3120–3478	Hypothetical protein	3044–3577	PH domain-containing protein	No
Contig 2	763–876	Hypothetical protein	791–1060	No significant similarity found	No
Contig 9	720–964	Hypothetical protein	616–954	Hypothetical protein	No

## Data Availability

The WGS of *Lp. plantarum* L125 and *Lp. pentosus* L33 are available at the NCBI Assembly database under the accession numbers JAIGOE000000000.1 and JAHKRU000000000.1, respectively.

## Supplementary Materials

The following supporting information can be downloaded at https://www.mdpi.com/article/10.3390/microorganisms11102553/s1, Table S1: ANI of *Lp. plantarum* L125 and *Lp. pentosus* L33 with members of their respective species; Table S2: Unaligned genomic regions of *Lp. plantarum* L125 organized in contigs and blasted against the “nucleotide collection (nr/nt)” and the “RefSeq Genome Database (refseq_genomes)” databases; Table S3: Unaligned genomic regions of *Lp. pentosus* L33 organized in contigs and blasted against the “nucleotide collection (nr/nt)” and the “RefSeq Genome Database (refseq_genomes)” databases; Table S4: Primer sets designed for the detection of *Lp. plantarum* L125 and *Lp. pentosus* L33 based on the unique genomic regions; Table S5: Unintended products generated by the primer pairs designed to detect *Lp. plantarum* L125 and *Lp. pentosus* L33 in other bacteria.
