# Supplementary material for: Development of a Multiplex PCR Assay for Efficient Detection of Two Potential Probiotic Strains Using Whole Genome-Based Primers
Source: Microorganisms. 2023 Oct 13;11(10):2553. doi: 10.3390/microorganisms11102553 (PMC10609308; doi:10.3390/microorganisms11102553)
Supplement: Supplementary file 1 [file microorganisms-11-02553-s001.zip › Table S5.pdf]

Table S5: Unintended products generated by the primer pairs designed to detect *Lp. plantarum* L125 and *Lp. pentosus* L33 in other bacteria.

Primer code	Unintended products (Size in bp)
L125 6.2F/R	*Massilia cavernae* K1S02-61 (2125)
L125 10.16F/R	*Paenibacillus silvestris* 5J-6 (3537)<br>*Maridesulfovibrio zosterae* DSM 11974 (2525)<br>*Eoetvoesiella caeni* DSM 25520 (254)<br>*Desulfosarcina cetonica* JCM 12296 (1956)<br>*Pedobacter foliorum* LMG 31463 (2279)<br>*Neobacillus fumarioli* NBRC 102428 (3192)
L125 12.1F/R	*Moorella sulfitireducens* SLA38 2 (2996)
L33 2.2F/R	None
L33 6.5F/R	*Tetragenococcus koreensis* KCTC 3924 (149)<br>*Desulfonatronovibrio magnus* AHT22 LZ23 (591)<br>*Deinococcus ficus* CC-FR2-10 (3190 & 2059)<br>*Gramella crocea* YB25 23 (1750)<br>*Gracilibacillus salitolerans* SCU50 (1120)<br>*Leptolyngbya ohadii* IS1 (790)<br>*Lactiplantibacillus pentosus* KW1 (380)<br>*Geobacter sp.* DSM 9736 (3336)
L33 9.8F/R	*Mycobacterium shinjukuense* JCM 14233 (3176)<br>*Dyella flava* DHOC52 (3434)<br>*Pelagicoccus enzymogenes* NFK12 191 (3906)<br>*Alkaliflexus imshenetskii* DSM 15055 (3591)<br>*Taibaiella chishuiensis* CGMCC (1902)<br>*Candidatus Viridilinea mediisalina* Kir15-3F (3057)<br>*Planococcus donghaensis* DSM 22276 (1277)<br>*Cognaticolwellia aestuarii* CGMCC1.6965 (781)<br>*Mucilaginibacter roseus* LMG 28454 (947)<br>*Lactococcus hodotermopsidis* Hs30E4-3 (3451)<br>*Chryseobacterium manosquense* Marseille-Q2069 (2062)<br>*Leptospira montravelensis* 201800278 (2254)<br>*Levilactobacillus brevis* NSMJ23 plasmid pLBN-3 (245)
